# Imaging in Acute Abdominal Emergencies: A Narrative Review of Surgical Decision-Making and Outcomes

**DOI:** 10.7759/cureus.95554

**Published:** 2025-10-28

**Authors:** Satabdi Dutta, Ovijit Dey, Sumit Midya

**Affiliations:** 1 General Practice, Frimley Health NHS Foundation Trust, Camberley, GBR; 2 General Medicine, Frimley Health NHS Foundation Trust, Camberley, GBR; 3 General Surgery, Frimley Health NHS Foundation Trust, Surrey, GBR

**Keywords:** abdominal injuries/diagnostic imaging, abdominal pain/diagnostic imaging, acute disease, appendicitis/diagnostic imaging, diverticulitis/diagnostic imaging, intestinal obstruction/diagnostic imaging, tomography x-ray computed, ultrasonography

## Abstract

Acute abdominal pain remains a common cause of emergency presentations, and modern imaging has transformed its evaluation and management. The introduction of ultrasound, computed tomography (CT), and magnetic resonance imaging (MRI) is generally believed to have improved diagnostic accuracy, reducing unnecessary surgical exploration and enabling safer, more targeted interventions. This review summarizes current evidence on the role of imaging in conditions such as appendicitis, trauma, diverticulitis, and bowel obstruction, highlighting its impact on surgical outcomes and clinical pathways. Key issues include balancing diagnostic speed with concerns over radiation exposure, cost, and potential overuse, particularly in vulnerable populations. Emerging technologies such as low-dose CT, artificial intelligence, and point-of-care imaging are likely to further refine practice, while addressing global disparities in access remains an ongoing challenge. This review uniquely synthesizes recent guideline updates and outcome-based evidence to highlight how modern imaging has redefined surgical decision-making in acute abdominal emergencies.

## Introduction and background

Acute abdominal pain is one of the most common and challenging presentations in emergency medicine and general surgery. It encompasses a broad differential diagnosis ranging from benign self-limited conditions to life-threatening surgical emergencies. Rapidly identifying which patients need urgent intervention, such as appendectomy for appendicitis or laparotomy for peritonitis, while avoiding unnecessary surgery in those without a surgical lesion, has historically been difficult based on clinical assessment alone. In the pre-imaging era, surgeons often adopted a more aggressive diagnostic approach - epitomized by the maxim "when in doubt, cut it out" [1,2]. This strategy inevitably led to a substantial frequency of negative surgical explorations, wherein no surgical pathology was found. For example, before routine imaging, negative appendectomy rates of 15%-25% were commonly accepted as the cost of not missing an occasional appendicitis [1]. In trauma, exploratory laparotomy was once the default for suspected intra-abdominal injury, with reports of non-therapeutic laparotomy rates on the order of 30%-40% in the 20th century [3-6]. These unnecessary surgeries expose patients to operative risks and morbidity with no benefit, highlighting a critical need for better diagnostic precision.

Over the last few decades, medical imaging has revolutionized the evaluation of the acute abdomen [2]. Modalities such as ultrasonography, computed tomography (CT), and magnetic resonance imaging (MRI) now allow clinicians to "see inside" the abdomen with a high degree of accuracy before deciding on operative intervention. The result has been a profound improvement in diagnostic confidence and a dramatic decline in negative appendectomies and laparotomies worldwide [7]. At the same time, the ubiquity of imaging, particularly CT, has generated new dilemmas regarding radiation exposure, incidental findings, cost, and possible over-reliance on technology at the expense of clinical acumen [8]. International bodies and specialty societies have developed evidence-based guidelines to optimize the use of imaging in various acute abdominal conditions - yet these guidelines do not always agree, reflecting different healthcare contexts and priorities (for instance, reducing missed diagnoses vs. minimizing radiation).

This review aims to provide a narrative and guideline-based examination of the role of imaging in specific clinical presentations of acute abdominal pain - namely appendicitis, diverticulitis, small bowel obstruction, and abdominal trauma - with emphasis on radiologic findings and their impact on surgical outcomes. We will review four major acute surgical conditions - especially acute appendicitis, colonic diverticulitis, bowel obstruction, abdominal trauma, and perforated viscus - to assess how imaging has influenced their diagnosis and management For each condition, we summarize key evidence from meta-analyses and large studies regarding imaging accuracy and impact on outcomes (such as reductions in unnecessary surgery or changes in management strategies). We compare recommendations from important guidelines (e.g., the American College of Radiology (ACR) Appropriateness Criteria [9], the World Society of Emergency Surgery (WSES) consensus guidelines [10], and the UK’s NICE guidance [11]) to highlight areas of consensus and divergence. Crucially, we discuss ongoing controversies and challenges: for example, the risk of CT overuse in emergency departments, radiation risks in children and pregnant patients, the debate over ultrasound-first protocols vs immediate CT, and the role of MRI as a radiation-free alternative [11-14]. We also present quantitative data wherever possible - such as the magnitude of reduction in negative appendectomy rates attributed to imaging [7] - to underscore how imaging has tangibly altered surgical practice. Finally, we explore future directions in the field, including technological innovations like low-dose imaging techniques and artificial intelligence for image interpretation [15-17], as well as the imperative to address global disparities in access to imaging that leave some populations behind [18-20].

Through this analysis, we seek to provide clinicians and researchers with an up-to-date understanding of how imaging can be optimally integrated into acute abdominal pain evaluation to improve patient outcomes, while also recognizing the need for judicious use. In the sections that follow, we delve into each major pathology and theme in detail, adopting an analytical lens informed by the latest literature and high-level evidence. Importantly, the role of imaging is discussed in the context of pre-test probability and clinical suspicion, as imaging is not a screening tool for “abdominal pain in general” but rather a supplement to appropriate clinical evaluation.

Methods and scope

This is a narrative review. We conducted a targeted literature search of PubMed and Embase (2010-2025) using terms such as “appendicitis,” “diverticulitis,” “small bowel obstruction,” “abdominal trauma,” “CT,” “MRI,” and “ultrasound.” Guideline statements from NICE, ACR Appropriateness Criteria, and WSES formed the primary basis of discussion, supplemented with recent systematic reviews, randomized trials, and large cohort studies. Articles were selected for relevance to surgical decision-making, diagnostic accuracy, and outcome implications. We did not perform a formal Preferred Reporting Items for Systematic Reviews and Meta-Analyses (PRISMA)-based systematic review or risk-of-bias assessment.

## Review

Imaging for suspected acute appendicitis

Diagnostic Accuracy and Impact on Negative Appendectomy Rates

Acute appendicitis is the archetypal abdominal surgical emergency and a condition in which the impact of modern imaging has been particularly well studied. Appendicitis is common (lifetime risk ~8% for males, ~7% for females) [2] and classically presents with migrating right lower quadrant (RLQ) pain, but atypical presentations are not uncommon [9]. Missing the diagnosis can lead to perforation and sepsis, whereas proceeding to surgery without confirmation risks removing a normal appendix [10,21]. Thus, striking the right balance has always been challenging. Before the widespread use of imaging, surgeons often accepted a substantial negative appendectomy rate (NAR) as unavoidable - historically, around 15%-25% of appendectomies yielded a normal appendix [1]. This meant one in five (or more) patients underwent an unnecessary operation, with attendant anesthesia, surgical risks, and costs, in the name of not missing a true appendicitis.

The introduction of cross-sectional imaging (ultrasound and CT) has dramatically improved diagnostic accuracy for appendicitis and slashed the negative appendectomy rate [7]. Large contemporary series and database studies illustrate this clearly. For instance, an analysis of over 11,000 appendectomy patients in the American College of Surgeons NSQIP dataset (2016) found an overall NAR of 4.5%, with utilization of CT in 86% of cases and ultrasound in ~15%. Patients who had no preoperative imaging had an NAR of 19%, whereas those evaluated with CT had a much lower NAR (~2.5%). In fact, when an ultrasound suggested appendicitis, adding a confirmatory CT reduced the false-positive surgery rate to virtually zero (NAR 0.6%). These data underscore that modern imaging can reduce unnecessary appendectomies to under 5%, a remarkable improvement compared to the pre-imaging era [1,2]. The ACR appropriately notes that the routine use of imaging in appendicitis has decreased NAR from as high as 20%-25% to approximately 1%-3% in many centers [1,2]. Similarly, a meta-analysis by Krajewski et al. demonstrated that the use of preoperative abdominal CT is associated with significantly lower negative appendectomy rates [7]; importantly, they found that the slight delay from imaging did not lead to an increase in appendiceal perforation rates compared to immediate surgery [7].

Comparison of Imaging Modalities

The main imaging tools for appendicitis are ultrasonography (US) - particularly graded-compression ultrasound of the RLQ - and CT scanning. Each has advantages and drawbacks. Ultrasound is radiation-free, relatively inexpensive, and often readily available; it is the preferred initial test in children, adolescents, and pregnant women, given its safety profile [12-14]. A competent ultrasound exam can directly visualize an enlarged, non-compressible appendix or secondary signs like peri-appendiceal fluid. However, ultrasound is operator-dependent and can be limited by body habitus or bowel gas, with non-diagnostic rates reported as high as 20%-30% in some series. Recent meta-analyses also highlight this heterogeneity, with sensitivity ranging from 71% to 94% depending on operator expertise and patient population [12,22].

CT, on the other hand, offers excellent visualization of the appendix and surrounding structures with high sensitivity and specificity (often >95%) [9]. CT has proven ability to identify alternative diagnoses as well, which is valuable since not all RLQ pain is appendicitis. The downsides of CT are exposure to ionizing radiation and the need for contrast in many protocols (with attendant risks like contrast allergy or nephropathy). MRI has emerged as a third modality, particularly useful in pregnant patients with inconclusive ultrasound: MRI avoids radiation and has diagnostic performance approaching that of CT for appendicitis, although it is less accessible in acute settings and requires longer scan times [13,14]. MRI is increasingly utilized in some centers for pregnant patients and can visualize appendicitis and even distinguish uncomplicated versus [13,14]. Complicated cases with good accuracy. From an economic and logistical standpoint, ultrasound-first strategies are cost-effective where expertise is available, while CT-first approaches dominate in centers with rapid scanner access. MRI remains accurate but limited by cost and availability [9].

Guideline Recommendations

There is broad agreement that imaging should be utilized in most cases of suspected appendicitis to confirm the diagnosis before operative management, with the only possible exception being those with classic presentations in certain low-risk scenarios. Guidelines differ slightly in recommended algorithms.

The ACR Appropriateness Criteria (2018) strongly endorse CT as the first-line imaging for adult non-pregnant patients with suspected appendicitis, given its superior accuracy [9]. Contrast-enhanced CT of the abdomen/pelvis is rated the most appropriate study, reflecting U.S. practice where rapid CT access is commonplace. The ACR notes that MRI is an emerging alternative as technology advances, and that while ultrasound can be used, CT remains the gold standard for overall performance. In pregnant patients, the ACR recommends ultrasound as first-line and MRI as the next step if ultrasound is non-diagnostic, reserving CT only if MRI is not available or the diagnosis remains unclear [9].

The WSES 2020 Jerusalem guidelines for acute appendicitis take a more stratified approach based on clinical probability and patient factors [22]. They advocate the use of clinical scoring systems (such as the Appendicitis Inflammatory Response (AIR) score or Alvarado score) to categorize patients into low, intermediate, or high risk for appendicitis. For low-risk patients, WSES suggests that a low score may obviate the need for any imaging, potentially allowing safe discharge or observation without radiation exposure. Intermediate-risk patients (equivocal cases) should undergo imaging to confirm diagnosis; here, WSES recommends an ultrasound-first strategy, especially in younger patients, with CT or MRI as a second-line if the ultrasound is non-diagnostic [22]. High-risk patients (classic presentation with high scores) may be considered for prompt surgery without imaging, especially if under age 40, to avoid delay and radiation - though this is a weak recommendation and acknowledges that many centers will still image to be certain. Overall, WSES strongly promotes POCUS (point-of-care ultrasound) as an appropriate first-line tool in both adults and children, as available, and explicitly recommends using low-dose CT protocols (with intravenous contrast) if CT is needed after a negative or indeterminate ultrasound [15,22,23]. WSES also emphasizes special populations: in pregnancy, graded compression ultrasound is recommended first, followed by MRI if further imaging is required; in pediatrics, ultrasound is first-line and routine CT is discouraged to minimize radiation, with MRI or surgical evaluation as alternatives if US is equivocal [13,14,22].

NICE (UK) guidance and other European sources similarly encourage an ultrasound-led approach in children and young adults, particularly females, to rule out gynecological differentials and minimize radiation [12]. In practice, many UK hospitals obtain an ultrasound for most women of childbearing age with RLQ pain. If the ultrasound is positive for appendicitis, surgery proceeds; if negative or unclear but clinical suspicion remains, a CT is then performed, or the patient is observed closely. This conditional imaging strategy (US first, CT second if needed) has been shown to effectively reduce unnecessary CTs while still maintaining a low negative appendectomy rate [12]. Some European centers even incorporate MRI for adolescents or young adults when expertise and equipment allow, as MRI avoids radiation entirely and has excellent specificity [13,22].

The result of these imaging protocols is a convergence toward very low rates of negative appendectomy internationally. Large population studies demonstrate current NARs generally in the 2%-5% range in centers that employ routine imaging, compared to double-digit rates decades ago [7]. It is worth noting that while imaging has reduced unnecessary surgery, it has also enabled consideration of non-operative management (NOM) of appendicitis in certain cases. With CT, one can distinguish uncomplicated appendicitis (no perforation, no abscess) from complicated [10,21]. Recent trials (e.g., CODA trial) have explored treating uncomplicated appendicitis with antibiotics first [22]. Guidelines like WSES acknowledge antibiotics-alone as an emerging option for select patients, but emphasize that imaging is crucial to ensure the appendicitis is truly uncomplicated (no abscess or rupture) before attempting NOM [22]. Thus, imaging not only guides the decision to operate, but in some cases guides the decision not to operate (i.e., treat medically). This exemplifies how imaging has broadened management strategies.

Controversies in Appendicitis Imaging

Despite consensus on the value of imaging, debates continue on specific points. One issue is radiation exposure, particularly from CT. Appendicitis predominantly affects children and young adults, for whom radiation is of greater concern [15]. Estimates suggest that abdominal CT (with effective doses ~5 to 10 mSv for low-dose protocols, higher for standard-dose) could slightly elevate lifetime cancer risk, especially if done at a young age [15]. This drives the push for ultrasound-first approaches in pediatrics and pregnant patients [12-14]. Many pediatric centers have demonstrated protocols that maintain NAR <5% while reducing CT utilization by relying on ultrasound and clinical observation, reserving CT for only unclear or high-risk cases [12]. Moreover, technical innovations allow lower-dose CT without major loss of accuracy - for example, a landmark RCT showed that a low-dose CT (median ~2 mSv) was non-inferior to standard-dose CT for appendicitis diagnosis, yielding similar negative appendectomy rates (~3% in both groups) [15]. WSES explicitly recommends using contrast-enhanced low-dose CT in patients with suspected appendicitis after a negative ultrasound, reflecting this priority to minimize radiation while retaining CT’s diagnostic power [22].

Another debate is whether a subset of patients with classic presentations truly needs imaging at all. Some surgeons argue that in patients with a classic history and examination findings, such as a young male, proceeding directly to laparoscopic appendectomy can avoid the small radiation risk of CT and save time. WSES supports possibly skipping imaging in high-probability, young, otherwise healthy patients (particularly men, in whom differential is limited) [22]. However, in many modern practice settings, even those patients often get a quick imaging confirmation because CT is so readily available and accurate. A study from Washington State’s SCOAP consortium noted that increased imaging use over time was associated with both a decline in negative appendectomies and an increase in detecting alternative diagnoses, supporting the practice of routine imaging even in seemingly obvious cases [7]. The counterpoint is resource utilization - each CT has cost and throughput implications, and in an environment of overcrowded EDs, judicious use of imaging is needed. Additionally, overreliance on imaging could, in theory, delay surgery in a patient who is actually clear-cut (though evidence suggests that such short delays do not worsen outcomes in appendicitis) [7]. Thus, practice varies: some European centers with fast ultrasound access may triage to the OR based on clinical + ultrasound findings within a couple hours, whereas many US centers. Hospitals will perform an immediate CT at triage for nearly all adult appendicitis suspects, given the near-instant availability of scanners [22].

In summary, imaging has become integral to appendicitis diagnosis. It has virtually eliminated the once-common exploratory operation where no appendicitis is found, thereby improving patient safety and saving healthcare costs associated with unnecessary procedures. The key is using the right imaging modality for the right patient: ultrasound (and possibly MRI) for children, adolescents, and pregnant patients, standard or low-dose CT for most adults, and always mindful of balancing speed, accuracy, and safety [15]. The trend in appendicitis management is toward precision: confirm the diagnosis, stratify severity (simple vs. complicated, which imaging can show by the presence of perforation or abscess), and tailor treatment (surgery vs. antibiotics, open vs. laparoscopic approach, etc. ) accordingly [10,16,21]. All of this is predicated on high-quality imaging and is reflected in drastically improved outcomes, for example, contemporary negative appendectomy rates in the low single digits and appendicitis perforation rates that have not increased despite more selective surgery [10,21].

Future Directions

Low-dose CT is increasingly emphasized as a strategy to minimize radiation without compromising accuracy. Randomized trials demonstrate that median doses of ~2 mSv are non-inferior to standard protocols, with similar negative appendectomy rates [15]. Guidelines such as WSES recommend adopting low-dose CT, particularly after a non-diagnostic ultrasound [22].

Artificial intelligence is also emerging in appendicitis imaging. Recent work has shown promise for deep learning applied to CT scans: a 2025 study developed a three-dimensional detection model that achieved high diagnostic accuracy for appendicitis [16]. Another group combined radiomics with deep learning to differentiate complicated from uncomplicated appendicitis, achieving AUC values above 0.8 [24]. Reviews also highlight the rapid progress and remaining challenges of integrating ML into CT-based appendicitis diagnosis [17]. Although these approaches are not yet in routine clinical use, they represent potential future tools to support radiologists and surgeons in decision-making.

Imaging in acute colonic diverticulitis

Diagnostic Modalities and Accuracy

Acute diverticulitis of the colon (usually left-sided, sigmoid colon) is another abdominal emergency where imaging plays a central role in modern management. Diverticulitis can range from mild inflammation to life-threatening perforation with peritonitis [10,21]. Traditionally, the diagnosis was clinical, sometimes supplemented by contrast enema or ultrasound, and management decisions (hospitalization, antibiotics, urgent surgery) were made with some uncertainty [25]. Now, CT scanning is considered the gold standard diagnostic modality for diverticulitis, as it can confirm the diagnosis and simultaneously stage the severity of the disease.

Meta-analyses confirm CT as the most accurate modality, with pooled sensitivities around 94%-97% and specificities 95%-98%, but also show that ultrasound can achieve sensitivities of 84%-92% in experienced hands, particularly in European centers where it is more widely used [25].

CT-Based Classification Systems

Over the past few decades, CT has revolutionized diverticulitis care by enabling detailed anatomic assessment. The classic Hinchey classification (stages I-IV) was originally based on operative findings of perforation and abscess, but newer CT-based classifications have been developed to stratify diverticulitis on imaging [26]. For example, CT can differentiate uncomplicated diverticulitis (localized colon wall thickening and fat stranding with no abscess) from complicated diverticulitis (abscess, fistula, obstruction, or free perforation) [25,26]. It can measure abscess size and locate it (e.g., pericolic vs. pelvic), detect extraluminal air or contrast indicating micro-perforation, and reveal diffuse peritonitis if present. All this information guides management: small abscesses (<4 to 5 cm) may respond to antibiotics alone, larger abscesses often require CT-guided percutaneous drainage, and frank perforation with peritonitis mandates emergency surgery (usually a resection with diversion) [21]. CT findings thus directly inform whether non-operative management is feasible or surgical intervention is required, and if so, whether a percutaneous approach is an option. Moreover, CT helps exclude other diagnoses that mimic diverticulitis (such as colon cancer - in fact, CT can sometimes identify a mass or suspicious features prompting a follow-up colonoscopy after acute inflammation settles) [25,26].

Impact of Imaging on Management

WSES guidelines (2016, updated 2020) on acute left-sided colonic diverticulitis explicitly state that CT is the primary diagnostic tool and has become the standard of care in an acute setting [25,26]. They note that ultrasound can be used by experienced hands and is somewhat helpful in thinner patients to detect inflamed bowel and abscesses, but CT offers a more comprehensive evaluation. In practice, many centers will perform a contrast-enhanced abdominopelvic CT on any patient suspected of moderate to severe diverticulitis or if it’s a first episode. Mild, recurrent cases in known diverticular disease might be treated empirically without imaging [27], but even that approach is waning because up to 10%-15% of presumed “uncomplicated diverticulitis” actually harbor complications or an alternate diagnosis that CT would clarify [25,26]. The precision of CT has allowed the development of various classification schemes (e.g., modified Hinchey, Neff, Ambrosetti, and the newer WSES CT grading) that correlate with outcomes and guide therapy [25]. For instance, the presence of an abscess on CT (Hinchey I or II) typically means antibiotics ± drainage, whereas Hinchey III (purulent peritonitis) or IV (fecal peritonitis) means emergency surgery [26].

Given that many patients experience recurrent diverticulitis, CT dose reduction is highly relevant. Low-dose CT protocols have been validated for diverticulitis with accuracy comparable to standard CT, significantly lowering radiation exposure [26].

With imaging stratification, the management of diverticulitis has shifted. In the past, many patients with diverticulitis (even without perforation) were taken to surgery after a couple of episodes or if they failed to settle quickly [10,21]. Now, non-operative management is first-line for the majority of cases - largely because CT can confirm the absence of generalized perforation [27]. Uncomplicated diverticulitis is routinely managed with antibiotics (and there is even emerging evidence questioning the need for antibiotics in very mild cases) [27]. Even some complicated cases, like a small abscess, can resolve with antibiotics alone [27]. CT-guided percutaneous drainage of larger abscesses (≥3 to 5 cm) is a major advance that often obviates immediate surgical intervention, turning an emergency surgery into an elective one or no surgery at all [25,26]. This technique relies entirely on imaging: interventional radiologists use CT (or ultrasound) to place a drain into the abscess, controlling sepsis and allowing the acute inflammation to subside, after which an elective colon resection may be done if indicated. Multiple studies and WSES guidelines endorse percutaneous drainage for accessible diverticular abscesses, with reported success rates typically 70%-90% in avoiding urgent surgery for that episode [26].

Emergent surgery is therefore mostly reserved for the severest spectrum (diffuse peritonitis or clinical failure of non-op management) [28]. Even when surgery is undertaken, imaging helps plan the operation: knowing if there is a distal obstruction or multiple abscesses can influence whether a primary anastomosis is attempted or a stoma is made [28]. In the elective setting, patients who have had episodes of diverticulitis will usually undergo a colonoscopy (to exclude malignancy) once healed, but CT in the acute phase already provides a preview, for example, if CT raises suspicion of a stricture or tumor, that accelerates further evaluation [25,26].

​​​​​*Outcomes*

The widespread use of CT has correlated with improved outcomes in diverticulitis, although it’s harder to quantify than for appendicitis. One specific outcome is a reduction in unnecessary colostomies or emergency colectomies [28]. In earlier decades, some patients without clear diagnostic imaging ended up in the OR with diagnostic uncertainty (for example, an elderly patient with diffuse abdominal pain might get exploratory surgery to find the cause, and a diverticular abscess could lead to resection and colostomy). Now, CT can identify localized abscesses that can be drained without surgery, sparing the patient an urgent colostomy [28]. A retrospective analysis would likely show fewer emergency surgeries for diverticulitis and more initial non-operative treatments since the advent of routine CT, with no increase in complication rates - in fact, probably a decrease in acute perforations because CT catches patients before they worsen [28]. Additionally, mortality from diverticulitis emergencies (e.g., perforation with sepsis) has improved over the last two decades, coinciding with widespread CT use that enables earlier diagnosis and targeted intervention [28].

Guideline-wise, the WSES 2020 update indicates that in immunocompetent patients with Hinchey III (purulent peritonitis), a trial of non-operative management may be considered in the absence of diffuse feculent peritonitis, particularly if CT shows only localized air and fluid, though this remains controversial and case-by-case [26]. This is a significant change from earlier dogma that all perforated diverticulitis needed surgery, possible only because CT allows careful selection of patients [25,26]. The European Society of Coloproctology (ESCP) and others similarly emphasize CT to direct management, and even suggest that for a first episode of uncomplicated diverticulitis, hospitalization might not be necessary if CT confirms the limited nature (some patients can be treated with oral antibiotics, outpatient if reliable) [25,26].

Ultrasound and MRI

While CT is dominant, it’s worth noting that ultrasound can diagnose diverticulitis with decent accuracy when performed by skilled operators [25]. Transabdominal ultrasound may show bowel wall thickening >5 mm and hyperechoic fat around a sigmoid colon segment, and can sometimes visualize an abscess. It is commonly used in some parts of Europe (e.g., by gastroenterologists or surgeons themselves) and can be the first imaging in young patients or pregnant patients [11]. MRI is not routine for diverticulitis but is an option for patients where CT is contraindicated (severe contrast allergy, pregnancy) [11]. MRI has high sensitivity for diverticulitis and can differentiate abscess vs. phlegmon, though access and speed are issues.

While CT offers unparalleled anatomic detail, its dominance partly reflects availability and convenience rather than inherent superiority in all settings. Meta-analyses show that outcomes can be comparable in centers with strong ultrasound expertise, highlighting that operator skill and local resources should guide the choice of first-line imaging [25].

In summary, imaging - particularly CT - is indispensable in acute diverticulitis for confirming the diagnosis and guiding treatment. It has enabled a tailored, less invasive management paradigm: most patients avoid emergency surgery and its attendant morbidity unless imaging shows they truly need it. The accuracy of CT (often reported >95% for diverticulitis diagnosis) means that clinicians can treat confidently and observe or intervene appropriately [25]. Current consensus holds that any moderate to severe presentation, atypical case, or failure to improve promptly should be imaged with CT if not done initially [26]. With CT-based staging, the era of one-size-fits-all management has given way to individualized care - an abscess can be drained, a contained perforation managed with antibiotics, and only diffuse peritonitis requires immediate surgical resection [21,28]. This approach has reduced patient morbidity (fewer ostomies, fewer emergent operations) and allowed many to recover without surgery. The improved outcomes are implicit in the evolution of practice, supported by guideline recommendations that strongly favor imaging confirmation of diverticulitis in any ambiguous or significant case [25,26].

Future Directions

Future directions in diverticulitis imaging include refinement of CT protocols and integration of advanced technologies. Non-contrast CT has recently been shown to be non-inferior to contrast-enhanced CT for diagnosis, raising the possibility of reduced radiation and contrast exposure in selected patients [29]. In parallel, artificial intelligence is being explored to help differentiate acute diverticulitis from mimics such as colon carcinoma, with early models demonstrating promising accuracy [30]. Finally, prospective radiology reviews emphasize the need for structured reporting systems and standardized classification schemes to improve communication between radiologists and surgeons [31].

Imaging in small bowel obstruction

Small bowel obstruction (SBO) is another common surgical emergency, often due to postoperative adhesions, hernias, or other intra-abdominal pathology [32-34]. Patients classically present with cramping abdominal pain, vomiting, distension, and inability to pass stool or flatus. In SBO, the pivotal management decision is usually whether to treat conservatively (bowel rest, decompression, fluids) or proceed to surgery - largely hinging on signs of bowel strangulation (ischemia) or an unresolved mechanical blockage [32-34]. Imaging has become critically important in this decision process, with CT now considered the preferred modality to evaluate SBO in most circumstances [35].

Diagnostic Modalities and Accuracy

Traditional vs. modern evaluation: Historically, diagnosis of SBO relied on abdominal X-rays (plain radiographs), which can show air-fluid levels and dilated loops suggestive of obstruction [32,33]. While useful, X-rays are relatively insensitive (particularly for partial obstructions) and cannot reliably identify the cause or presence of strangulation. Contrast studies (ingestion of water-soluble contrast followed by timed X-rays) have been used both diagnostically and therapeutically to judge if contrast passes through the obstruction, indicating likely resolution. These still have a role - indeed, meta-analyses show that if oral contrast given for an SBO does not reach the colon within 24 hours, surgery is very likely needed, whereas if it does, the obstruction will probably resolve without surgery [33]. However, CT has surpassed these methods in providing comprehensive information in one study [32].

Timing of CT vs. Contrast Challenge

Regarding timing, two pragmatic strategies are used: (2) early CT to characterize cause, transition point, and ischemia; and (2) an initial water-soluble contrast challenge (with plain radiographs) in clinically stable patients with presumed adhesive SBO, reserving CT for failure of contrast progression or clinical deterioration. Evidence supports the predictive value of the contrast challenge (lack of colonic transit within 8-24 hours predicts need for surgery), while guidelines favor early CT in most cases because of its ability to identify closed-loop/strangulation and alternative etiologies [33,34].

CT in SBO: Modern multi-detector CT can confirm the diagnosis of obstruction, often identifying the transition point (the exact location where bowel caliber changes from dilated to collapsed) and frequently revealing the cause (e.g., adhesive band, hernia, tumor, intussusception) [33]. Modern multidetector CT demonstrates high diagnostic performance for SBO, with pooled sensitivity approximately 91-95% and specificity 89-96% for detecting obstruction; for predicting ischemia/strangulation, reported sensitivity is ~82-87% with specificity ~92-96%. CT also identifies a transition point in most cases (often >90%), which is critical for operative planning [33,35]. Importantly, CT can detect signs of strangulation or ischemia: bowel wall thickening, lack of bowel wall enhancement, mesenteric edema, poor perfusion, pneumatosis (air in the bowel wall), or portal venous gas are ominous findings [33]. Even subtler signs like mesenteric twisting (the *whirl sign*) indicate a volvulus or closed-loop obstruction that urgently needs surgery [34]. A systematic review noted that CT has around 90% accuracy in predicting which SBO patients have strangulation and will require urgent surgery [35]. This prognostic ability is critical - the mortality risk in SBO comes largely from missed strangulation leading to infarcted bowel. By using CT, surgeons can confidently identify high-risk cases early [36].

WSES (Bologna) guidelines for adhesive SBO explicitly state that CT scan is the preferred imaging technique if there is any doubt about diagnosis or to assess the need for urgent surgery, due to its ability to exclude other causes and to evaluate for complications [33]. They assert that while plain X-rays should be done initially (and often are, as a quick screening test), a CT should follow in most cases except the most straightforward, because CT provides the anatomical and etiological details plain films cannot. Additionally, CT can guide nonoperative management decisions: for instance, if CT shows a partial obstruction with no signs of ischemia and perhaps a small amount of contrast passing, one might manage nonoperatively; but if CT shows closed-loop obstruction or strangulation signs, one goes straight to surgery [33,34]. The guidelines and many experts suggest that the threshold for obtaining a CT in SBO should be low, given the high stakes of missing a strangulation and the availability of effective treatment if identified [33,34].

Impact on Management and Outcome

The advent of routine CT for SBO has a few notable outcomes.

Reduced delay to necessary surgery: In the past, if a patient was observed nonoperatively but actually had an undiagnosed strangulation, it could result in gangrene and a worse outcome by the time surgery was finally done. CT helps avoid this by prompting earlier intervention when needed. Some retrospective studies have found that the introduction of CT reduced the incidence of delayed operations and bowel gangrene, thereby lowering complication rates and length of stay for surgical SBO cases (though exact quantification varies) [33,36].

Avoiding unnecessary surgery: Conversely, CT can identify patients who do not need immediate surgery. Many SBOs (especially adhesions) will resolve with conservative management. Traditional teaching was to observe 24-72 hours as long as there were no peritonitic signs, but this can be anxiety-provoking without knowing what the obstruction looks like. CT that shows only distended loops but with contrast reaching the colon, or an adhesive obstruction without a closed loop, can give confidence to persist with nonoperative treatment safely. Studies have shown that water-soluble contrast CT followed by an abdominal X-ray at 24 hours is a reliable method: if contrast hasn’t passed, surgery is indicated (predictive accuracy >95%) [33,34]. If it has passed, continued nonoperative management is likely to succeed. This protocol both speeds up surgery when needed and prevents premature surgery when not needed. As a result, overall fewer exploratory surgeries may be performed for SBO that would have resolved on their own, and those who do go to surgery are better selected (i.e., a higher proportion have ischemic or refractory obstruction that truly necessitates it). The WSES summary recommends exactly this approach: CT with oral water-soluble contrast early in management, followed by an abdominal X-ray at 8-24 hours; failure of contrast to progress is an indication for surgery [33,34].

Identification of cause: CT has ~95% specificity in identifying a transition point and can often hint at cause [36]: for example, a small bowel loop herniating through an abdominal wall defect (incisional or inguinal hernia) will be apparent, guiding the surgical approach. If a mass is causing obstruction, CT will detect it, which not only directs surgery but also aids oncologic planning if it’s a tumor. If inflammatory bowel disease or intussusception is the cause, CT will clarify that as well. In adhesive obstructions, adhesions themselves are not seen, but CT excludes other causes and can sometimes see a beak or kink suggestive of an adhesive band. Knowing the cause preoperatively can reduce surprises and allow better operative strategy (for instance, planning laparoscopy vs. open surgery) [35,36].

Special situations: In pregnant patients with suspected SBO, or when radiation is to be avoided, MRI can be a substitute; it can show obstruction and ischemia signs reasonably well, though it is not as validated as CT in this context [32,33]. Ultrasound has limited penetration for full SBO assessment in adults, but can sometimes show dilated loops and peristalsis in high-grade obstruction, and may be more feasible in pediatric or pregnant patients [32,33]. The WSES guidelines mention ultrasound and MRI as potentially useful in specific scenarios (operator-dependent ultrasound in experienced hands, and MRI for pregnancy or when CT is unavailable) [32,33]. They highlight that ultrasound is widely available even in low-income settings and can detect free fluid or suggest strangulation (like free fluid plus dilated loops might indicate a higher risk), but acknowledge that CT is the preferred modality in general.

Guideline Recommendations

The WSES adhesive SBO guideline (2018) conclusions reinforce: “CT scan with oral water-soluble contrast is the preferred initial imaging in ASBO” [33]. If a patient is known to have adhesions and has had recent imaging excluding other pathology, one might go straight to a therapeutic Gastrografin trial without a new CT, but any uncertainty merits a CT [35]. They also advise that if the diagnosis is certain and no urgent signs, one can do the contrast follow-through without an upfront CT (for example, a patient with prior surgeries, classic SBO presentation, and an abdominal X-ray already diagnostic of SBO could be managed with a contrast study alone) - but again, that requires certainty that there’s no need for urgent surgery, which usually comes from either prior imaging or very careful clinical judgement [33].

Outcome metrics: One possible outcome of more widespread CT use in SBO is reduced mortality and bowel loss from missed strangulation; historical surgical series support reduced mortality when CT-identified transition zones are promptly addressed [36]. Olausson et al. also report that in contemporary cohorts where CT is nearly ubiquitous, delays to operation decrease and outcomes remain acceptable [37]. Moreover, a prospective study by Zielinski et al. identified certain CT findings (mesenteric edema, the *small-bowel feces *sign absence, etc.) that predicted need for surgery; having such criteria helps triage patients appropriately [34]. The key point is that with imaging, surgical intervention for SBO can be targeted to those who truly need it, thus avoiding the morbidity of surgery in others and preventing catastrophic delays in those who do need surgery.

Imaging in abdominal trauma

Abdominal trauma, particularly blunt abdominal trauma, has seen one of the most significant shifts in management philosophy attributable to advances in imaging. In the past, exploratory laparotomy or Diagnostic peritoneal lavage was the default for many trauma scenarios due to limited diagnostic options - surgeons often had to operate to definitively diagnose internal injuries (a principle encapsulated in the old *triad* for trauma: mechanism, positive diagnostic peritoneal lavage, and go to OR) [3-6]. This led to a high rate of non-therapeutic laparotomies (where no injury requiring repair is found) with all their attendant morbidity. Now, with fast and accurate imaging, trauma surgeons can often avoid surgery in favor of nonoperative management for many injuries, and only operate when imaging confirms a surgical lesion [6]. The result is far fewer unnecessary laparotomies and a paradigm shift toward selective management.

*Focused Assessment with Sonography for Trauma* *Ultrasound*

The first big imaging advance was the Focused Assessment with Sonography for Trauma (FAST) exam - a rapid ultrasound exam of the pericardium and peritoneal spaces for free fluid (blood). Introduced in the 1990s, FAST quickly became a staple of initial trauma evaluation. It is a bedside, radiation-free tool that can be done within minutes upon patient arrival. A positive FAST (free fluid in abdomen) in an unstable patient is an indication for immediate laparotomy, as it implies internal hemorrhage. FAST thus reduced the need for diagnostic peritoneal lavage (an invasive test) and expedited decisions. FAST can be repeated serially as well without harm, helping to monitor a patient’s status. However, FAST only detects fluid and gives no detail on injury location or severity, so by itself it doesn't guide non-operative vs. operative management beyond the binary decision. Recent studies confirm that FAST has modest sensitivity (ranging from 65%-85%) but very high specificity (>95%), meaning it is excellent for ruling in intra-abdominal injury in unstable patients but inadequate to rule out injury in stable ones [38]. It is highly useful in blunt trauma when the question is emergent laparotomy vs not (especially in hypotensive patients), and in penetrating trauma to check cardiac tamponade or significant hemoperitoneum [38,39].

CT Scanning in Trauma

Perhaps the greatest *game changer* has been CT imaging of the trauma patient. Modern trauma protocols in high-resource settings involve performing a whole-body CT (also called *pan-scan*) from head to pelvis in blunt trauma patients who are stable enough to undergo scanning [3,4]. The sensitivity of CT for internal injury is extremely high - it can visualize injuries to solid organs (liver, spleen, kidneys), hollow viscus, vascular structures, etc., with detailed grading [40]. CT allows clinicians to truly know what injuries are present and their extent without surgical exploration. This information has enabled a huge shift: many injuries once thought to mandate surgery are now safely managed without it, as long as the patient remains hemodynamically stable [3,4,40]. For example, splenic and liver lacerations - historically often managed by surgical removal or repair - are now treated nonoperatively in the majority of cases, with success rates >95% for low-grade injuries and even 80-90% for many high-grade injuries, provided patients are monitored in ICU and have ready access to interventions if needed. This selective approach is underpinned by the American Association for the Surgery of Trauma (AAST) organ injury grading system, which stratifies liver, spleen, and kidney injuries from I (minor) to V (severe vascular disruption), with most grades I-III now managed nonoperatively. This change is directly attributable to CT’s ability to accurately grade organ injuries and detect active bleeding. If CT shows a contained splenic laceration with no active arterial contrast extravasation, a surgeon can be confident to observe the patient, whereas in the pre-CT era, they might have operated based on mechanism and suspicion alone [40].

Whole-body CT (pan-scan) remains debated. While randomized and observational studies suggest a survival advantage in polytrauma, a 2024 meta-analysis reported no significant mortality difference (odds ratio (OR) 0.94, 95% confidence interval (CI) 0.83-1.06) between pan-scan and selective CT, indicating that benefits depend on patient selection [41]. Pan-scan can also reveal incidental findings, add financial cost, and expose patients to ~20 to 30 mSv radiation per scan [8]. Moreover, unstable patients may not tolerate transfer to the scanner and require operative or bedside decision-making. Contemporary prospective studies show that in stable patients, pan-scan frequently alters management (~64% of cases), but its use should be individualized [42].

## Conclusions

This narrative review highlights how advances in imaging have transformed the evaluation and management of acute abdominal emergencies. Routine use of modalities such as ultrasound, CT, and MRI has reduced negative appendectomy rates, lowered non-therapeutic laparotomy rates, improved risk stratification, and supported wider adoption of non-operative management where safe.

These benefits must be interpreted in context: imaging outcomes vary with operator expertise, local resources, and patient factors, while risks of radiation exposure, incidental findings, and costs remain. Clinically, this underscores the importance of using imaging as a complement to sound clinical judgment rather than a replacement for it. Practical strategies include: prioritizing ultrasound or MRI in children and pregnant patients, adopting validated low-dose CT protocols, using clinical scoring systems to guide when imaging is appropriate, and reserving whole-body CT for hemodynamically stable trauma patients.

Looking forward, innovations such as artificial intelligence, structured reporting, and wider use of point-of-care ultrasound offer opportunities to further enhance accuracy, minimize harm, and improve access-particularly in resource-limited settings. For clinicians, the implication is clear: when applied judiciously, imaging is a powerful tool that enables safer, more precise, and more individualized surgical decision-making.
